# Comparison of the Agilent, ROMA/NimbleGen and Illumina platforms for classification of copy number alterations in human breast tumors

**DOI:** 10.1186/1471-2164-9-379

**Published:** 2008-08-08

**Authors:** LO Baumbusch, J Aarøe, FE Johansen, J Hicks, H Sun, L Bruhn, K Gunderson, B Naume, VN Kristensen, K Liestøl, A-L Børresen-Dale, OC Lingjærde

**Affiliations:** 1 Department of Genetics, Institute for Cancer Research, Norwegian Radium Hospital, Rikshospitalet University Hospital, 0310 Oslo, Norway; 2 Department of Pathology, Norwegian Radium Hospital, Rikshospitalet University Hospital, 0310 Oslo, Norway; 3 Biomedical Research Group, Department of Informatics, University of Oslo, P.O. Box 1080 Blindern, 0316 Oslo, Norway; 4 Faculty Division The Norwegian Radium Hospital, University of Oslo, Oslo, Norway; 5 Cold Spring Harbor Laboratory, Cold Spring Harbor, New York 11724, USA; 6 Agilent Technologies, Inc., 5301 Stevens Creek Blvd, Santa Clara, CA 95052, USA; 7 Illumina, Inc., 9885 Towne Centre Drive, San Diego, CA 92121, USA; 8 Department of Oncology, Norwegian Radium Hospital, Rikshospitalet University Hospital, 0310 Oslo, Norway; 9 Centre for Cancer Biomedicine, University of Oslo, Oslo, Norway

## Abstract

**Background:**

Microarray Comparative Genomic Hybridization (array CGH) provides a means to examine DNA copy number aberrations. Various platforms, brands and underlying technologies are available, facing the user with many choices regarding platform sensitivity and number, localization, and density distribution of probes.

**Results:**

We evaluate three different platforms presenting different nature and arrangement of the probes: The Agilent Human Genome CGH Microarray 44 k, the ROMA/NimbleGen Representational Oligonucleotide Microarray 82 k, and the Illumina Human-1 Genotyping 109 k BeadChip, with Agilent being gene oriented, ROMA/NimbleGen being genome oriented, and Illumina being genotyping oriented. We investigated copy number changes in 20 human breast tumor samples representing different gene expression subclasses, using a suite of graphical and statistical methods designed to work across platforms. Despite substantial differences in the composition and spatial distribution of probes, the comparison revealed high overall concordance. Notably however, some short amplifications and deletions of potential biological importance were not detected by all platforms. Both correlation and cluster analysis indicate a somewhat higher similarity between ROMA/NimbleGen and Illumina than between Agilent and the other two platforms. The programs developed for the analysis are available from .

**Conclusion:**

We conclude that platforms based on different technology principles reveal similar aberration patterns, although we observed some unique amplification or deletion peaks at various locations, only detected by one of the platforms. The correct platform choice for a particular study is dependent on whether the appointed research intention is gene, genome, or genotype oriented.

## Background

Microarray technology has become a powerful tool for many scientific and diagnostic applications. In cancer research the detection of genomic aberrations is crucial for associating copy number changes with cancer phenotypes or critical genes. For array Comparative Genomic Hybridization (array CGH), several methods and platforms have been developed (see reviews [1,2]). Microarray copy number detection systems differ in their probe origin (BAC, cDNA or oligonucleotides [3-6]), production (spotting, polymerization or microbeads), gene density (coverage of probes per gene or physical intercept), hybridization (digestion, hybridization to reference), and labeling technique (single or two-color systems). Laboratories are often required to evaluate the diverse microarray formats, considering different biological questions, experimental designs, material restrictions, and resolutions or data processing challenges. Comparability and reproducibility of results have always been important issues. Hence, it is important to evaluate microarray platforms not only based on their production characteristics but also using a variety of analytical and statistical methods. A comparative analysis of expression platforms has previously been performed for gene expression measurements [7-10]. However, to our knowledge this is one of the first publications validating different array CGH formats using tumors as material.

In this report, we compare three major DNA microarray platforms: The Agilent Human Genome CGH Microarray 44 k, the ROMA/NimbleGen Representational Oligonucleotide Microarray 82 k, and the Illumina Human-1 Genotyping 109 k BeadChip. Oligonucleotide probes used for the Agilent array cover both coding and non-coding sequences, and most reporters are located in genes (gene oriented arrangement). Oligonucleotides in the ROMA/NimbleGen technology are based on *Bgl *II cutting sites, hence reporters are more or less randomly distributed across the entire genome providing a detailed picture of the structure and organization of the complete genome (genome orientated arrangement). The Illumina platform on the other hand provides a dense, exon-centric view of the genome (genotyping arrangement). The three platforms were tested with a set of 20 primary breast tumor samples. The samples are part of a larger cohort of stage I and II primary tumors [11], including several distinct expression subtypes expected to present with a number of common aberrations for human breast cancer [12]. Results achieved were validated using different graphical and statistical methods, many performed with the CGH-Explorer analysis tool [13]. One goal of our study was to investigate to what extent platforms of different nature and design perform differently in terms of detecting aberrant structures, regarding both size and amplitude of copy number changes. The results of the analysis were evaluated to investigate whether the number of probes, density distribution, probe localization, sensitivity and aberration calling method had any effect on the overall performance of the platform. Overall, we found that all platforms included in this study give a similar general picture of the DNA rearrangements in the tumors, including genomic instability profiles, although some details differ substantially.

## Results

### Whole genome analysis reveals overall similarity between platforms

**Figure 1:**
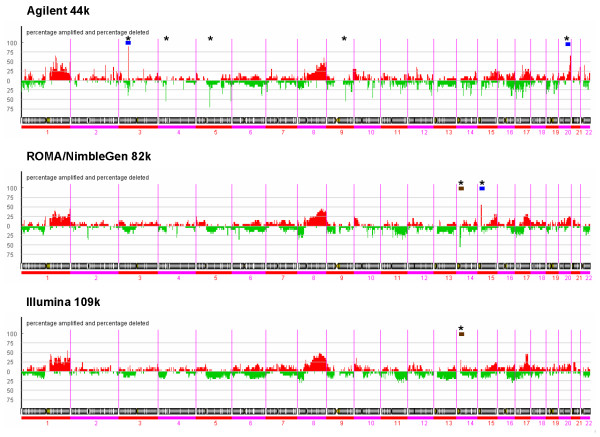
**Frequency plot of copy number aberrations**. Copy number changes of 20 breast tumors are illustrated using the Piecewise Constant Fit (PCF) method in the CGH-Explorer program. Amplified regions are marked red and deleted regions are marked green. The proportion of calls (all samples combined, and deletions and amplifications combined) was adjusted to 12% (for details see Methods). For every platform, specific high frequency (over 50%) small amplification or deletion peaks are visible (marked by stars *). For the Agilent platform: amplifications at 3p, and 20q, deletions at 4p, twice at 5q, and 9q; for the ROMA/NimbleGen platform: amplifications at 15q and deletions at 14q; the Illumina platform shows lower frequency peaks, but picked up a unique amplification at 14q not seen by the other platforms. (Indicated with blue bars are regions, for which additional information is provided in Figure 7 and in Additional files 8, 11, and 12. Further are indicated with brown bars regions, for which additional information is in Additional files 9 and 10).

**Figure 2:**
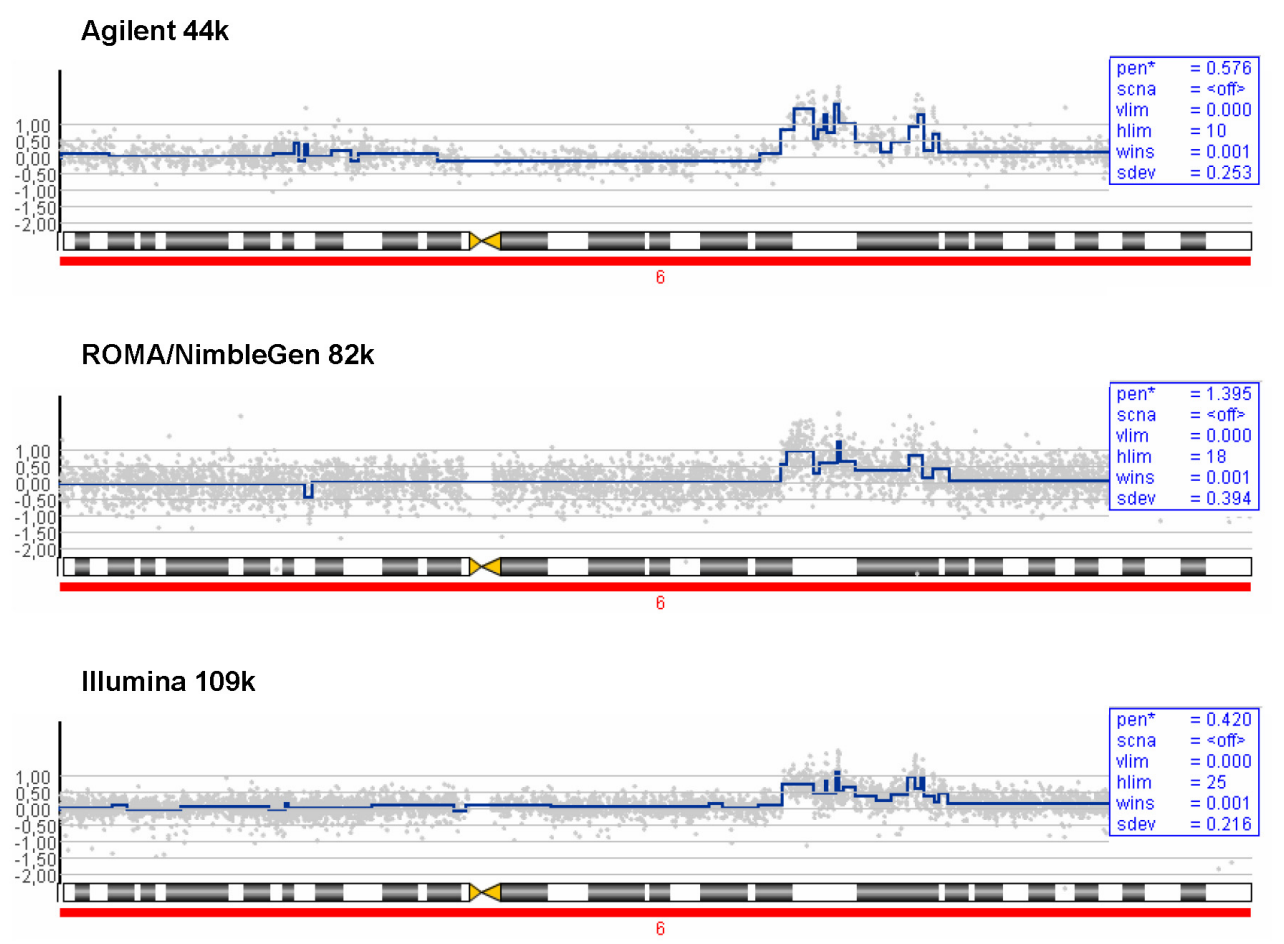
**Piecewise constant regression for chromosome 6**. Detection of genomic aberrations using the PCF method for all platforms, shown for chromosome 6 for patient sample 146. Despite the great similarities in the overall size, structure and amplitude of aberrations, several minor variations are seen.

The overall pattern is similar for all three platforms, as confirmed by the results displayed in Figure 1. Here, the overall frequency of amplification and deletion events in the tumor samples was estimated for the three platforms using the PCF (Piecewise Constant Fit) algorithm (see Methods). Analysis of copy number changes using a method based on a different principle (ACE – Analysis of Copy Number Errors [13]) showed the same overall pattern (see Additional file 1). All platforms detect the same previously described common aberrations [14-16], like high frequency of amplification of chromosomal regions 1q, 8q, 17q and deletions at 16q and 17p (Figure 1 and see Additional file 1). Figure 2 illustrates that the platforms reveal similar results with respect to amplification peaks, though with some minor differences in amplitude height and/or number of events, amplification region size or pattern.

**Figure 3:**
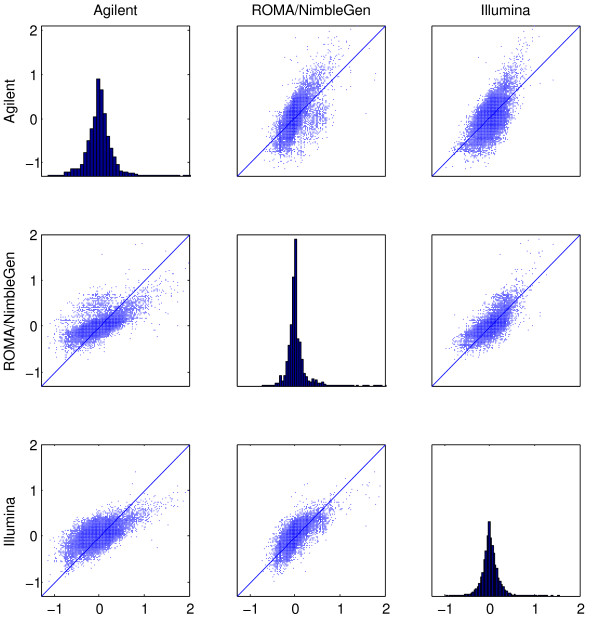
**Scatterplot matrix comparing platforms**. Scatterplot matrices for 20 human breast cancer samples are shown for each pair of platforms. The scatterplot matrices are based on the vectors of PCF values. Panels off the diagonal: For each of the 20 samples, PCF values are found for one platform were plotted against values found for another. Abscissa values are for the Agilent 44 k (left column), ROMA/NimbleGen 82 k (middle column) and Illumina 109 k (right column) platforms. Ordinate values are for the Agilent 44 k (top row), ROMA/NimbleGen 82 k (middle row) and Illumina 109 k (bottom row) platforms. Panels on the diagonal: The distribution of the PCF filtered log ratios for all 20 breast cancer samples combined, for the Agilent 44 k (top left panel), ROMA/NimbleGen 82 k (middle panel) and Illumina 109 k (lower right panel) platforms, respectively.

**Figure 4:**
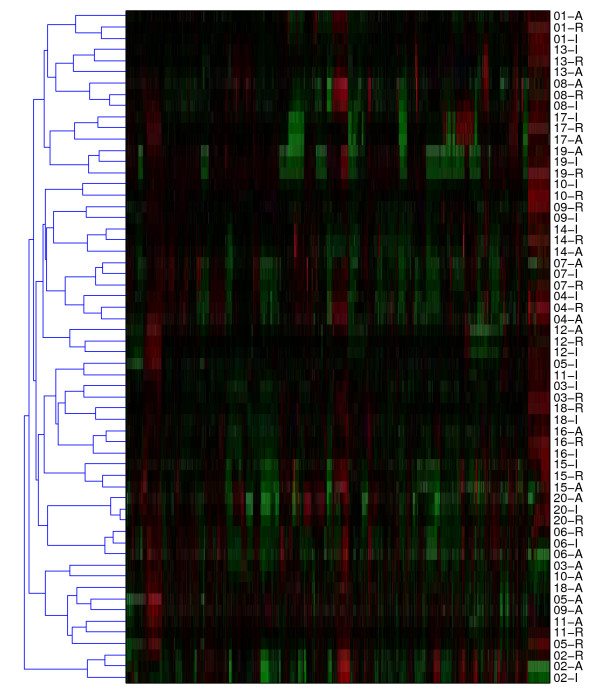
**Clustering of 60 arrays representing 20 tumors on 3 platforms**. The figure shows the result of clustering the PCF estimated log ratios of 3 × 20 arrays obtained from the 3 platforms used on the 20 tumor samples (for PCF vector calculation see Methods). The samples were clustered using Spearman correlation and average linkage (A = Agilent 44 k, R = ROMA/NimbleGen 82 k, or I = Illumina 109 k). For 14 of the 20 tumors, the three platforms are clustered together at the lowest possible cluster level, for 4 of the remaining tumors, Illumina and ROMA/NimbleGen are clustered together.

Figure 3 shows histograms for the distributions of the PCF-fitted copy numbers and scatterplots comparing all three platforms. There is strong correlation between platforms, though with a notable difference in scale between Agilent and the two other platforms, in accordance with what other authors have reported on cell lines [17]. Computing correlation coefficients between the platforms for the tumor samples confirms this picture, with median Pearson correlation of 0.77 for Illumina versus ROMA/NimbleGen and close to 0.6 for Agilent versus the other two platforms (see Additional file 2). We also applied Total Least Squares (TLS) regression to fit regression lines with zero intercept to the data in Figure 3. This analysis yielded a median slope of 0.47 (IQR = 0.45) for Agilent versus ROMA/NimbleGen, 0.55 (IQR = 0.26) for Agilent versus Illumina, and 0.99 (IQR = 0.52) for ROMA/NimbleGen versus Illumina. In Figure 4 the PCF estimated log ratios are used to cluster the 60 cases obtained from the 3 platforms used on the 20 tumor samples. For 14 of the 20 tumors, the three platforms are clustered together at the lowest possible cluster level and for 4 of the remaining tumors Illumina and ROMA/NimbleGen cluster together.

**Figure 5:**
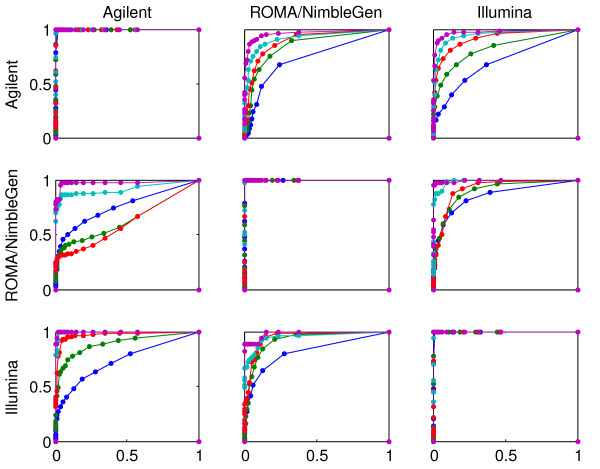
**ROC curves for aberration calling**. ROC curves for the classification of 2936 uniformly spaced genomic loci into three categories (a) deletion, (b) normal and (c) amplified. Using a fixed threshold T across the genome, every locus was called as a deletion if the PCF value was less than -T and as an amplification if the PCF value was larger than T. For every panel in the figure, one of the platforms is chosen as the correct classification (Agilent in row 1, ROMA/NimbleGen in row 2, and Illumina in row 3). The ROC curves show the ability of each of the remaining two platforms to mimic this correct classification. There are four curves in each panel, corresponding to the thresholds T = 0.25, 0.50, 0.75, 1.00 (shown in blue, green, red and cyan, respectively) used to define the correct classification. The points on a ROC curve correspond to different choices of T for the other platform under consideration.

Figure 5 shows ROC curves comparing the platforms pairwise with respect to aberration calling. Not surprisingly, the curves reveal an increasing correspondence as the threshold for calling an aberration is increasing. This is confirmed by inspecting the Area Under Curves (AUCs) for the ROC analysis (see Additional file 3). A somewhat better correspondence for Illumina versus ROMA/NimbleGen than for Agilent versus the other platforms is seen.

Additional file 4 shows the degree of concordance between the platforms with respect to the classification of probes as amplifications, deletions or normal. The table is based on one particular selection of detection thresholds, the relative size of the thresholds reflecting the relative scale of the log ratios of the different platforms. In all platforms, the majority of probes are classified as being normal (i.e. neither amplified nor deleted). Probes that are called as amplified (or deleted) on one platform are most often called likewise or as normal on the platform considered for comparison. Opposite decisions, in the sense that one platform calls an event as amplification and the other platform calls a deletion, are very rare. Hence, in terms of the direction of aberrations, the platforms are in large agreement with each other. Nevertheless, there is a substantial proportion of probes that are called on one platform and not on another. Note that the detection thresholds used here are not optimized with respect to the number of concordant classifications of probes.

**Figure 6:**
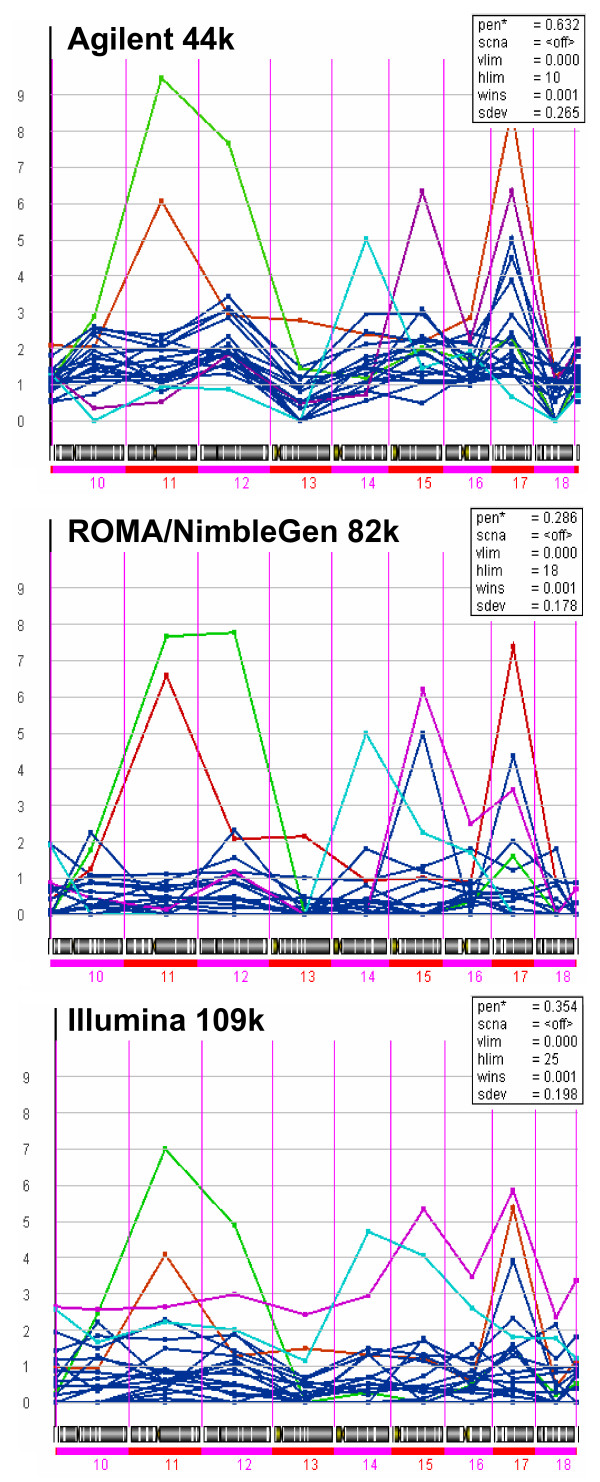
**Instability index for chromosome 10 to 18**. The instability index, indicating regions of clustered, narrow peaks of chromosomal rearrangements located at a chromosome arm or entire chromosome, was calculated for all samples for all chromosomes. Sample 085 shows highest instability index for chromosome 11 and 12 for all platforms, followed by sample 053 in chromosome 11. For chromosome 14 sample 263 has the highest instability index, for chromosome 15 sample 148. Small differences are seen for chromosome 17, here, the Agilent and ROMA/NimbleGen platforms indicate that sample 053 has the highest index, however, using the Illumina platform, sample 148 has the highest value. (Samples with high instability index (>2) are colored: Sample 053 = red, sample 085 = green, sample 148 = violet, and sample 236 = blue).

Instability indices, scoring the presence of localized regions of clustered, narrow amplification peaks on a chromosome arm, were determined (see Methods) for each platform in each of the 20 tumor samples. In all platforms, very high instability indices for chromosome 10 to 18 were found for patient 085 (green) on chromosome 11 and 12, and for patient 053 (red) on chromosome 17 (Figure 6). Additionally, high instability indices were detected for patient 053 on chromosome 11, on chromosome 15 for patient 148 and on chromosome 14 for patient 263.

### Differences between the various platforms due to gene density or other factors

Despite the general consistency of the platforms, specific variations in frequency and/or in aberration length are visible (see PCF analysis in Figure 1 and the detailed plot for chromosome 8 in see Additional file 5). Most of these differences are due to variance in probe location or density. Probe location is depending on design (evenly distributed probes or clusters of several probes per gene), which may be based on an automatic or manual strategy. Reporters are not evenly spaced in any of the platforms, accounting for differences in genome structure and natural variance in gene density. The distribution of probes for the three different platforms is illustrated for the complete genome (see Additional file 6) and in a close-up for chromosome 8 (see Additional file 7). Of all platforms, ROMA/NimbleGen comes closest to a uniform probe distribution, while Agilent shows high local variation of number of reporters, particular for areas in 1q, 3p, 6p, 11q centromeric, 12q centromeric, 16, 17 and 19. The Illumina platform shows high-density islands of reporters at 6p, 11p telomeric, 12q centromeric, 17p telomeric,19p, and 19q (see Additional file 6).

Interestingly, platform specific small high frequency amplification or deletion peaks were found, as indicated by (*) in Figure 1. Many were nearby the centromeric or telomeric regions. The Agilent platform shows unique small high frequency aberrations of amplification at 3p and of deletions at 4p, twice at 5q, and 9q, and a larger amplification increase towards the telomere of 20q. ROMA/NimbleGen exhibits unique small high frequency deletion at 14q and an amplification at 15q. Increasing the aberration detection sensitivity, some extra platform dependent unique small high frequency amplifications or deletions are observed, e.g. at 14q for the Illumina platform.

**Figure 7:**
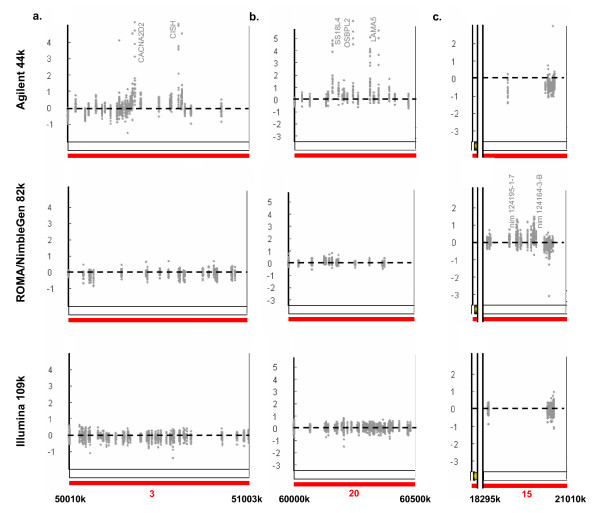
**Platform differences in narrow frequent amplification peaks**. a.) The Agilent platform identifies a small amplification peak at chromosome 3, between 50.36–50.64 Mb (for exact probe localization, aberration calling, reporter coverage and gene identification see Additional file 8). The area is called as amplified using the Agilent platform due to two strongly amplified reporters covering the genes CACNA2D2 (H.s. calcium channel, alpha 2/delta subunit 2) and CISH (H.s. cytokine inducible SH2-containing protein). b.) A larger unique amplification region is detected by the Agilent platform at chromosome 20q, between 60.00–60.50 Mb. Reporters covering SS18L4, OSBPL2, and LAMA5 are highly amplified, causing this region to be called as amplification in the Agilent platform. Reporters in the other platforms either miss these genes or are located nearby at different positions (for exact probe localization, aberration calling, reporter coverage, and gene identification see Additional file 12). c.) The ROMA/NimbleGen platform detects a unique small amplification peak at chromosome 15, between 18.40–20.40 Mb (for details of exact probe localization, aberration calling, reporter coverage and gene identification see Additional file 11), while the Agilent platform has one single reporter and Illumina platform provides only 2 reporters in this specific area, and are therefore unable to detect amplifications in this region.

We suspect some of the observed unique differences in copy number aberration calling to be of biological importance. We therefore examined examples of these features (Figure 1, indicated by blue or brown bars over chromosomes 3, 14, 15, and 20) in more detail (in close-ups in Figure 7 and by providing detailed information see Additional files 8, 9, 10, 11, 12): At chromosome 3, between position 50.36–50.64 Mb in over 75% of all samples the Agilent platform identifies a region with small amplifications (Figure 7a, for exact probe localization see Additional file 8). Two strongly amplified reporters covering the genes CACNA2D2 (H.s. calcium channel, alpha 2/delta subunit 2) and CISH (H.s. cytokine inducible SH2-containing protein) cause the amplification detection in the Agilent platform. The Agilent platform further detects a larger unique region with amplifications for telomeric region 20q, between 60.00–60.50 Mb (Figure 7b). Genes in this region (see Additional file 12) include SS18L1 (H.s. synovial sarcoma translocation gene on chromosome 18-like), OSBPL2 (H.s. oxysterol binding protein-like 2) and LAMAY5 (H.s. laminin, alpha 5), a gene of potential importance as it is found in the intrinsic gene list used for the classification of breast cancer subtypes [18]. Further, a unique short deletion is detected in over 50% of all samples using the ROMA/NimbleGen platform for the centromeric region of chromosome 14, at 19.00–20.00 Mb (see Additional file 9), covering a region of genes including CCNB1IP1 (H.s. cyclin B1 interacting protein 1), APEX1 (H. s. nuclease multifunctional DNA repair enzyme 1) and TEP1 (H. s. telomerase-associated protein 1). Interestingly, an adjacent unique region detected solely by the Illumina platform in chromosome 14 stretches from 21.20–22.00 Mb (see Additional file 10). Unique small amplification peaks exclusively detected by one of the platforms are likely to be due to differences in reporter density, as seen for the ROMA/NimbleGen platform at chromosome 15, between 18.40–20.40 Mb (Figure 7c). This centromeric peak is densely covered by 19 ROMA/NimbleGen reporters: However, the Agilent platform has a single reporter and Illumina platform provides only 2 reporters for this area (see Additional file 11).

## Discussion

Microarray-based comparative genomic hybridization allows the construction of high-resolution maps of genome-wide copy number alterations. Array CGH enables localization of genomic aberrations in tumors, identification of critical genes, and classification of chromosomal changes [1], indicating susceptibility or activation of tumor initiation and progression [19]. Different arrays have been used for CGH-studies starting with cDNA-, followed by BAC-, and more recently by high density oligonucleotide-arrays [3-5,20,21]. Only few comparisons of the various array CGH platforms have been performed and those we are aware of [17,22,23] were based on other platforms and/or cell lines rather than on tumor data. For example, in [17] the focus is primarily on reproducibility, signal-to-noise ratio and resolution differences.

We compared the Agilent Human Genome CGH 44 k oligonucleotide Microarray, the Representational Oligonucleotide Microarray ROMA/NimbleGen 82 k array, and the Illumina SNP-CGH Human-1 (109 k) BeadChip array platforms using human breast tumor samples. Whole genome analysis of called copy number alterations reveals a great overall similarity and strong correlation between platforms. However, in concordance with [17] we detected a notable difference in the scale of the log ratios between Agilent and the two other platforms. In their cell line study with known relative copy number, [17] found markedly higher signals for the Agilent 44 k than for the high-resolution ROMA/NimbleGen 1500 k. Numerically, the factors detected in their study correspond well to the factor of about 0.5 found in our TLS analysis of our tumor samples. Agilent 44 k CGH arrays have a gene oriented arrangement being enriched particularly for cancer relevant genes with local high variation of number of reporters. The combination of these two features may be the reason for the high number of specific small high frequency amplification or deletion peaks particular in the Agilent platform (Figure 1 and see Additional file 6). The ROMA technology, invented at Cold Spring Harbor Laboratory and by NimbleGen [20], is based on representative oligonucleotide probes designed for fragments of the human genome sequence, which are more or less randomly distributed across the genome. The ROMA/NimbleGen arrays provide a gene-independent arrangement of the structure of the complete genome at a high resolution. The bead-based Human-1 109 k arrays from Illumina provide an exon-centric view of the genome through their 109 k SNP markers, of which 70% are located in gene exons or within 10 kb of transcripts. This platform employs an allele-specific primer extension assay using two probes (bead types) in one color channel to score Single-Nucleotide Polymorphisms (SNPs) [24,25].

It is important to be able to distinguish between real copy number variation and variation related to the technical processing and analysis of the arrays. Various programs, web-based tools and statistical software packages are available for exploring and analyzing array CGH data, e.g. [13,26,27], with different accuracy for the estimation of aberrations callings [26]. A major advantage of the aberration calling method used here (PCF), as compared to many other aberration calling algorithms, is that its mathematical form makes it easy to adjust parameters to adapt to the specific platforms to obtain an appropriate comparison.

In comparing platforms, one should be aware that higher density of probes not necessarily implies an improved effective resolution. The effective resolution of a platform, defined as the smallest genomic range for which a reliable classification can be made with respect to aberration status (deleted, normal or amplified), clearly depends on the platform's probe density and on the platform's SNR (Signal-to-Noise Ratio). Let D denote the absolute change in true intensity log-ratio corresponding to the smallest copy number alteration that we want to be able to detect, and suppose the observed intensity log-ratios are normally distributed around the true log-ratio with standard deviation SD. Then, we may define SNR = D/SD. To be able to detect the smallest alteration while controlling the Type I and Type II error rates, the number of probes in the region must exceed a number that scales as the inverse square of SNR. Thus, the effect of doubling the signal-to-noise ratio is comparable to that of increasing the probe density by a factor of four. Of course, an added effect of increasing the probe density, as opposed to improving the SNR, is that local aberration details may be revealed that are partly or completely absent in lower resolution scans.

The content and performance characteristics of a particular platform influence its applicability for a certain study type. We conclude that for gene detection and gene-oriented research the Agilent or the Illumina platform are to be preferred. On the other hand, the ROMA/NimbleGen approach, showing a compact picture of the entire genome structure, is the method of choice for exploration of the various mechanisms leading to different types of genomic instability such as Chromosomal Instability (CIN) or Microsatellite Instability (MIN). The Illumina SNP-CGH arrays can be utilized for detecting LOH and allelic ratios in detecting aberrations. We are aware that several newer platforms with increased reporter numbers exist. However, the aim of this study was to assess to what extent platforms of different nature and design can result in differences in detection of some copy number aberration patterns.

In general, when comparing samples hybridized to different platforms the following steps should to be taken, supposing the data have been normalized according to the platform used: Fit a regression model suitable for copy number data, such as PCF, to obtain estimated log ratios for a desired set of genomic loci. Parameters (for PCF: The penalty parameter and the lower limit on the size of a plateau) should reflect the probe density and noise level. If some samples have been hybridized to both (all) platforms, frequency plots (like Figure 1) should be used to estimate the difference in scale. Then perform aberration calling on the basis of the above fit, compensating for scale differences when estimates are available. Finally, caution should be taken when comparing the small-scale structure of aberrations, since our analysis indicates that the similarities found by different platforms in the higher-level structure are not necessarily accompanied by correspondence in the detailed structure.

## Conclusion

Using 20 breast tumor samples and adjusted analytical methods, we observed high overall concordance between the three platforms evaluated, despite substantial differences in the platform composition. Both correlation and cluster analysis indicate a somewhat higher similarity in results obtained by ROMA/NimbleGen and Illumina than between Agilent and the other two platforms. Some short amplifications or deletions of potential biological importance were revealed by only one of the platforms. Detailed examination of these sites indicated that the discrepancy was mainly due to the density and spatial distribution of probes, and other platform specific features. Further studies are needed to verify on the potential biological importance of the sites. The correct platform for a particular study is dependent on the research intention, whether it is gene, genome, or genotype oriented, and on region or location of interest. A complete genomic tiling array including high density gene oriented reporters may be the ultimate goal for the study of genomic alterations in cancer.

## Methods

### Patient samples

From May 1995 to December 1998, 920 patients referred for surgical treatment of breast cancer were included from five different hospitals in the Oslo region in a large study on detection of isolated tumor cells in bone marrow [28]. From theses 920 patients, tissue samples from 20 breast carcinomas were selected for this study. All 20 breast carcinomas contained >40% tumor cells, the majority of the tumor specimens represent tumor size T1/T2, node status N0/N1 (9/11) and histological grade 2 or 3. The 20 samples have been classified into five clinically relevant tumor subclasses [18]. Tumor DNA was extracted using an ABI 341 Nucleic Acid Purification System (Applied Biosystems) according to the manufacturer's protocol.

### Agilent platform

Agilent's Human Genome CGH Microarray 44 k contains 44,255 in situ synthesized 60-mer probes (3,877 controls) designed for studying copy number changes and representing most of the known or predicted human genes. The probes are, after manufacturers description, enriched for cancer relevant genes representing both coding and non-coding sequences on the chromosomes [29]. Experiments using Agilent arrays were performed at the Department of Genetics at The Norwegian Radium Hospital, Oslo, Norway using female human genomic DNA (Promega) as reference and following the Agilent recommended standard protocol (see Additional file 13). The arrays were scanned using an Agilent scanner, data extraction, filtering and normalization were conducted using the Feature Extraction software version A.7.5.1 (Agilent Technologies). The CGHAnalytics program version 3.4.40 (Agilent technologies) was used to export the array CGH data for usage in other analytic programs.

### ROMA/NimbleGen platform

The Representational Oligonucleotide Microarray Analysis (ROMA) has been developed at Cold Spring Harbor Laboratory [20]. The procedure is based on representative oligonucleotide probes designed by analyzing *Bgl *II restriction fragments of the human genome sequence. Approximately 85,000 70-mer probes are randomly combined on a single chip, providing a more or less even distribution across the human genome. ROMA experiments were prepared and analyzed at the Cold Spring Harbor Laboratory, New York, USA using male reference DNA (CHP-SKN-1 = 46, XY male) and following the standard ROMA/NimbleGen protocol (see Additional file 13). Arrays were immediately scanned using an Axon GenePix 4000b scanner (pixel size as set to 5 *μ*m). The GenePix Pro 4.0 software was used for identification and quantification of probe intensities. Measured intensities without background subtraction were used to calculate ratios. ROMA/NimbleGen data were normalized using an intensity-based lowness curve fitting algorithm [30].

### Illumina platform

The SNP-CGH experiments were performed using the Infinium™ I assay on the Human-1 Genotyping BeadChip representing 109,365 loci (~109 k) [21,24]. Each allele is represented by two unique beads, having an average of 30-fold redundancy per unique bead. The BeadChips are constructed by attaching 50-mer probes to 3 *μ*m-diameter beads, which are randomly assembled onto the chips containing ~3 *μ*m diameter wells. In addition to the 50-mer probe sequence, a ~30-mer address sequence is present on each bead to allow identification of each bead by decoding [24]. The Illumina experiments were prepared and analyzed at Uppsala University, Uppsala, Sweden. For the Illumina Human-1 BeadChip (109,365 loci) samples were prepared and processed according to the manufacturer's protocol (see Additional file 13). Signal detection was conducted using the Illumina BeadArray Reader (Infinium I FastScan scanner protocol) while identification of bead positions and raw-data extraction were performed using the BeadScan software. Following data acquisition, data from patient blood samples (of 112 corresponding blood-tumor pairs) were subjected to clustering using the algorithm supplied in the BeadStudio application. These clusters were furthermore applied to the tumor arrays, and manual review of peripheral GenCall (GC) and Cluster Separation (CS) scores was performed. After clustering and QC-review, we extracted the log R-ratios for the tumor data. This ratio results from dividing the normalized R-value (observed) by the expected normalized R-value [21].

### Statistical methods and analytical tools

### The PCF algorithm

The PCF algorithm is an extension of the *Potts filter *method described by Winkler *et al*. [31] and seeks the best possible fit to the data using one or more constant plateaus. Let *D* = {(*x*_*i*_, *y*_*i*_), *i *= 1,..., *n*} be copy number data for one chromosome in one individual, where *a *= *x*_1 _≤ *x*_2 _≤ ⋯ ≤ *x*_*n *_= *b *are the locations of the probes along the chromosome and *y*_1_,..., *y*_*n *_are the corresponding log-transformed copy number ratios. Then the PCF filtering algorithm computes the solution $\hat{z}=({\hat{z}}_{1},...,{\hat{z}}_{n})$ to the penalized optimization problem

$$\underset{z}{\min}H_{\lambda }(z|y)\equiv \underset{z_{1},...,z_{n}}{\min}\left\{\sum_{i=1}^{n}{\left(y_{i}-z_{i}\right)}^{2}+\lambda \cdot \#\{i|z_{i}\neq z_{i+1}\}\right\},$$

the first term in braces being the goodness of fit and the second term being a penalty proportional to the number of discontinuities (jumps) in the function. The constant *λ *> 0 controls the trade-off between the two terms. Observe that the transformation (**y**, *λ*) → (*σ***y**, *σ*^2^*λ*) induces a corresponding transformation $\hat{z}\to \sigma \hat{z}$ of the solution. Letting the penalty coefficient be *λ *= *τσ*^2 ^where *τ *> 0 is a constant and *σ*^2 ^is the variance of the log ratios, the number of discontinuities or their locations will not be scale dependent. To compare different platforms we select a platform-independent value of *τ *(say *τ *= 9) and for any particular chromosome and array let *λ *= *τ*${\hat{\sigma }}^{2}$ where ${\hat{\sigma }}^{2}$ is the estimated variance of the log ratios. The PCF algorithm used in this paper also allows the user to specify a lower limit on the size (number of probes) of a plateau in the piecewise constant function to be determined. To compensate for the platform differences in average probe density, the limit was set to 10 probes for Agilent, 18 for ROMA/NimbleGen and 25 for Illumina.

### Cross-platform copy number comparison

Several of the analyses in this paper involve the comparison of copy number measurements across platforms. As the actual measurement probes for one platform differ in number and genomic locations from that of another platform, some assumptions must be made about the copy number ratio between neighboring probes in order to carry out a meaningful comparison. The PCF algorithm provides a useful starting point, as it eliminates (or reduces) through smoothing the random variability owing to the measurement process, while at the same time it fits a piecewise constant regression function to the log ratios which is defined everywhere on the genomic range of the data. Specifically, the PCF solution $\hat{z}$ may be extended to a function defined on the whole range of the data:

$$\hat{f}(x)=\{\begin{array}{lll} {\hat{z}}_{1} & \text{if} & x_{1}\leq x\leq u_{1}\\ {\hat{z}}_{i} & \text{if} & u_{i-1}<x\leq u_{i}\\ {\hat{z}}_{n} & \text{if} & u_{n-1}<x\leq x_{n} \end{array}$$

where *u*_*i *_= (*x*_*i *_+ *x*_*i*+1_)/2. This means that solutions obtained for different platforms (with different probe locations) can be directly compared through interpolations of the PCF curves in identical genomic loci chosen to be identical for all platforms. For the analyses in this paper, the loci were chosen to be uniformly spaced across the whole genomic, with a distance of 1 Mb between neighboring loci (leading to a total of 2936 genomic loci in which the PCF function value was determined by the above interpolation formula). Thus, for every array a vector **z **of PCF function values of length m = 2936 was found and was used to produce scatterplots comparing different platforms, to cluster the arrays, to compare aberrations across platforms, etc.

### Aberration calling

To call aberrations in a tumor, a two-step procedure is applied to the log-transformed copy number ratios. First, a piecewise constant regression function is fitted to the log ratios, using the PCF algorithm described above. Next, a gene is called if the fitted regression value for the gene is above a certain positive threshold T (amplification call) or below -T (deletion call). To validate the results obtained from PCF, we also applied an unrelated aberration calling algorithm called ACE [13] to the data. Overall, the aberration patterns found by the two methods are very similar.

### ROC curves

To assess the degree of similarity between the aberration patterns found with different platforms, we consider the ability of each platform to mimic the other two platforms' classification of genomic loci. For any platform and any sample, we first apply the PCF algorithm to the log-transformed copy number ratios to fit a piecewise constant function. Let T be a fixed positive threshold. For 2936 genomic uniformly spaced genomic loci across the genome, we classify the locus as a deletion, if the corresponding PCF value is less than T, and as amplification if the PCF value is larger than T. Otherwise, the genomic locus is classified as normal. This is done for various choices of T, for every one of the 20 samples and for every platform. For any given pair of platforms, we consider the classification based on one platform as the correct (true) classification and calculate an ROC curve for the classification based on the other platform relative to the first one. The points on an ROC curve correspond to different threshold values T for the classification based on the second platform (T ranging from 0 to the maximal PCF value for that platform). Different ROC curves may be produced by varying the threshold used to define the correct classification.

### Total least squares regression

To investigate possible differences in scale between the log ratio measurements obtained for the different platforms, we fitted for every pair of platforms regression lines with no intercept to PCF interpolation values, obtained on a regular genomic grid consisting of 2936 loci. Total Least Squares regression was used for this purpose. While an ordinary least squares regression fit takes into account measurements errors in the dependent variable only, TLS also accounts for measurement errors in the independent variable. This leads to an improved estimate of the slope for data in cases, where both platforms are subject to substantial measurement error. Estimates of slopes were found separately for each array, and the median slope is reported together with a robust measure of spread called Interquartile Range (IQR), defined as the difference between the 3rd and 1st quartile of the 20 slopes.

### Detection of *firestorms*

Hicks *et al*. (2006) observed in some tumors what they referred to as a *firestorm *in the array CGH profile. A firestorm is the presence of at least one localized region of clustered, relatively narrow peaks of amplification, with each cluster being confined to a single chromosome arm [30]. In order to detect firestorms, we define a mathematical measure called a *firestorm index*, designed to quantify the degree of instability in a chromosome. As before, let *D* = {(*x*_*i*_, *y*_*i*_), *i *= 1,..., *n*} be the copy number observations for one particular chromosome and let $\hat{z}=({\hat{z}}_{1},...,{\hat{z}}_{n})$ be the piecewise constant fit found by the PCF algorithm described above. Suppose ${\hat{\sigma }}^{2}$ is an estimate of the variance of the data around the true mean. Select the points *x*_*i *_for which ${\hat{z}}_{i-1}\neq {\hat{z}}_{i}$ and at least one of the values ${\hat{z}}_{i-1},{\hat{z}}_{i}$ are outside the interval $0\pm 2\hat{\sigma }$, and denote these points *t*_1 _< ... <*t*_*d*_. Let *a*_1 _< ... <*a*_*d *_be the corresponding log ratios. For a window *w *spanning *s *bases of the chromosome (this paper uses *s *= 35 Mb), define

$$\gamma (\omega )=\frac{N(\omega )}{\hat{\sigma }}\sum_{t_{i},t_{i+1}\in \omega }\left|a_{i+1}-a_{i}\right|$$

where *N*(*ω*) is the number of turning points (i.e. local minima and maxima) in the sequence {(*x*, ${\hat{z}}_{i}$), *x*_*i *_∈ *ω*}. The firestorm index is defined as the maximum of *γ*(*ω*) as *ω *ranges over all windows that cover *s *bases of the chromosome.

## Authors' contributions

LOB: carried out the Agilent experiments together with JA, participated in the analytical method development, performed data analysis, and writing of the manuscript including preparation of the majority of the figures.  JA: carried out the Agilent experiments together with LOB, drafted the methods section, and critical reading of the manuscript.  FEJ: carried out the Illumina experiments, participated in the discussions and drafted the Illumina part of the methods section in the manuscript. JH carried out the ROMA/NimbleGen experiments, participated in the discussions. HS: provided support and discussion of the Agilent array experiments, and critical revision of the manuscript.  LKB: provided support and discussion of the Agilent array experiments, and critical revision of the manuscript. KG: provided support and discussion of the Illumina experiments, and critical revision of the manuscript. BN: provided the breast cancer material and clinical data, and critical revision of the manuscript.  VNK: participated in the design of this study, coordinated the Illumina collaboration, and critical revision of the manuscript.  KL: participated in the development of the analysis methods, as well as in discussing, writing, and critical reading of the manuscript.  ALBD: conceived the study, and participated in its design and coordination, participated in the discussions and critical reading of the manuscript.  OCL:  participated in the development of the analysis methods, as well as in discussing, writing, and critical reading of the manuscript.  

All authors read and approved the final manuscript.

## Supplementary Material

Additional file 1 **Whole genome analysis of copy number errors by ACE**. Illustrated are frequency plots for amplified (red) or deleted (green) regions for 20 breast cancer samples along the genome using copy number errors (ACE) analysis and graphical tools in the CGH-Explorer program [13]. ACE is less sensitive than PCF, but it detects well known amplification regions for chromosome 1, 8, and 17, in addition to an amplification increasing towards the telomere for chromosome 20, only detected in the Agilent platform. Click here for file

Additional file 2 **Platform correlation**. The three boxplots give the correlations between the platforms. Correlations are based on PCF values found for every array and for a regular genomic grid (see the Methods section on cross-platform copy number comparison), and the box plots shows the distribution of the 20 correlations found for every pair of platforms. Click here for file

Additional file 3 **Genomic aberration on chromosome 8 using PCF method**. Single plot graphical views for each of the 20 tumor samples are shown using the Piecewise Constant Fit (PCF) method in the CGH-Explorer program for detection of copy number changes. Amplifications are highlighted in red and deletions are marked with green with color intensity coding from -0.5 to 0.5 and an overall high similarity are seen (see Methods). Despite wide similarities some differences are also detected. For example, sample 031 shows an interrupted deletion at the p arm for the Agilent and Illumina platforms missing in the ROMA/NimbleGen platform, further sample 042 shows a low copy number gain of several regions on 8q by the ROMA/NimbleGen and Illumina platforms, missing in the Agilent platform. Click here for file

Additional file 4 **Gene density plot for the whole genome**. The distribution of probes along the complete genome is illustrated for the Agilent 44 k, ROMA/NimbleGen 82 k, and Illumina 109 k platforms with platform-dependent bandwidth selection (see Methods). Uneven distribution is seen for all platforms with areas of high density in certain chromosomes or chromosome arms. Click here for file

Additional file 5 **Probe density plot for chromosome 8**, The distribution of probes is illustrated for the complete genome for three different platforms with bandwidth size, Agilent = 10^-6^, ROMA/NimbleGen = 10^-6^, and Illumina = 10^-5 ^for example chromosome 8 (gene density for the entire genome is presented in Additional file 6). Click here for file

Additional file 6 Area Under Curve for ROC analysis for the three platforms Agilent 44 k, ROMA/NimbleGen 82 k, and Illumina 109 k. Click here for file

Additional file 7 A PCF curve was fitted to each array and interpolation values were obtained on a regular genomic grid consisting of 2936 loci (see Methods). Aberrations were called using the following thresholds of detection (see Methods), equal to T = 0.40 for Agilent, T = 0.19 for ROMA/NimbleGen, and T = 0.23 for Illumina. Shown are confusion matrices for every pair of classifications (Agilent versus ROMA/NimbleGen, Agilent versus Illumina, and ROMA/NimbleGen versus Illumina). Click here for file

Additional file 8 Copy number variations for chromosome 3, 50.30–50.70 Mbp. Listed are platforms Agilent 44 k (A), ROMA/NimbleGen 82 k (R), and Illumina 109 k (I), position in bp, clone ID, gene description and name. Click here for file

Additional file 9 Copy number variation for chromosome 14, 19.00–20.00 Mbp. Listed are platforms Agilent 44 k (A), ROMA/NimbleGen 82 k (R), and Illumina 109 k (I), position in bp, clone ID, gene description and name. Click here for file

Additional file 10 Copy number variation for chromosome 14, 21.20–22.00 Mbp. Listed are platforms Agilent 44 k (A), ROMA/NimbleGen 82 k (R), and Illumina 109 k (I), position in bp, clone ID, gene description and name. Click here for file

Additional file 11 Copy number variation for chromosome 15, 18.40–20.40 Mbp. Listed are platforms Agilent 44 k (A), ROMA/NimbleGen 82 k (R), and Illumina 109 k (I), position in bp, clone ID, gene description and name. Click here for file

Additional file 12 Copy number variation for chromosome 20, 60.00–60.50 Mbp. Listed are platforms Agilent 44 k (A), ROMA/NimbleGen 82 k (R), and Illumina 109 k (I), position in bp, clone ID, gene description and name. Click here for file

Additional file 13 Details of standard protocols for the Agilent Human Genome CGH Microarray 44 k, ROMA/NimbleGen Representational Oligonucleotide Microarray 82 k, and Illumina SNP-CGH Human-1 109 k. Click here for file
