# Supplementary figures and images for: Comparison of the Agilent, ROMA/NimbleGen and Illumina platforms for classification of copy number alterations in human breast tumors
Source: BMC Genomics. 2008 Aug 8;9:379. doi: 10.1186/1471-2164-9-379 (PMC2547478; doi:10.1186/1471-2164-9-379)

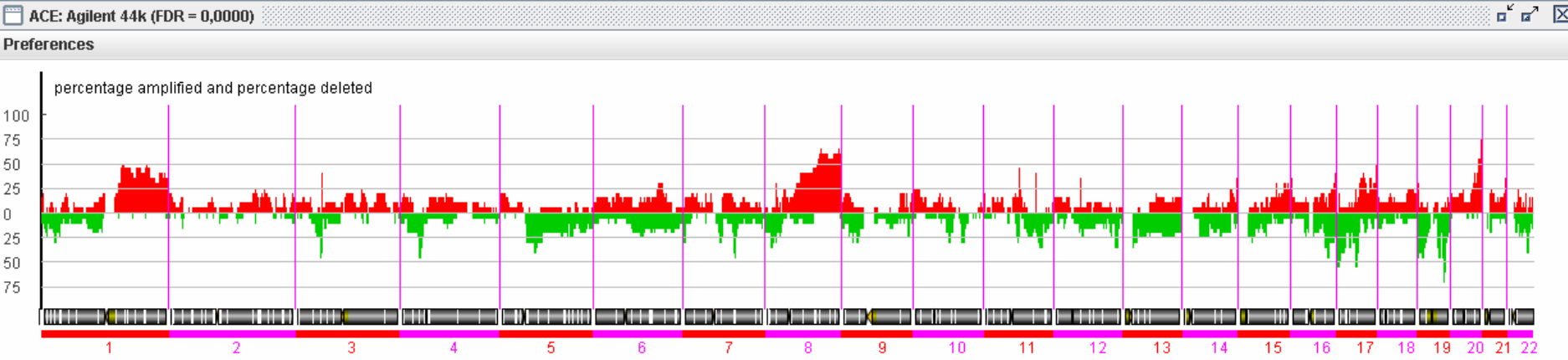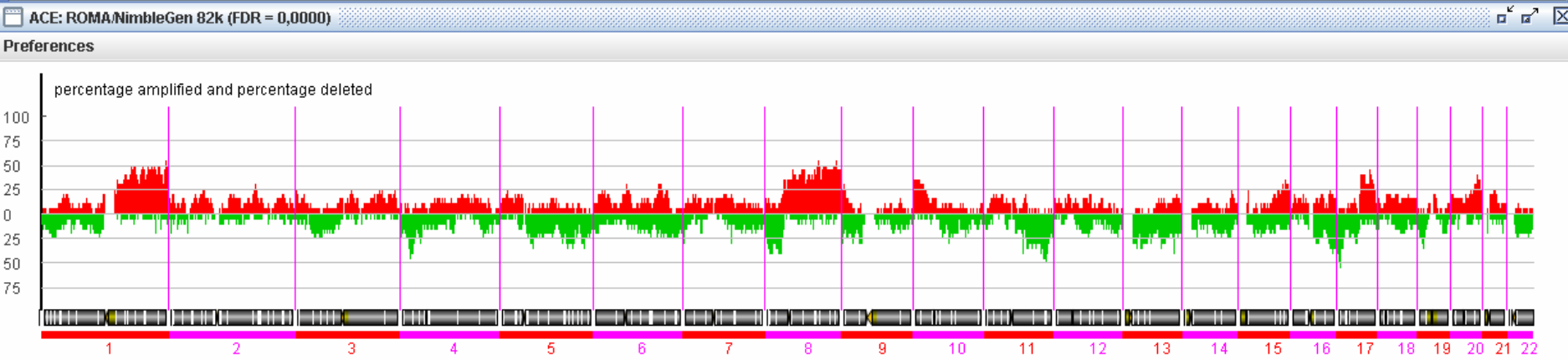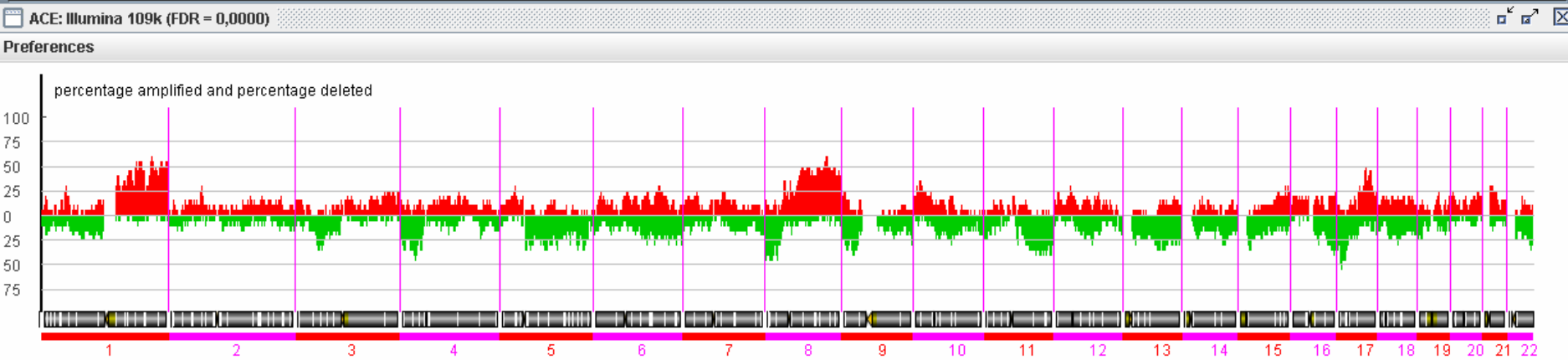

Supplement: Additional file 1 — Whole genome analysis of copy number errors by ACE. Illustrated are frequency plots for amplified (red) or deleted (green) regions for 20 breast cancer samples along the genome using copy number errors (ACE) analysis and graphical tools in the CGH-Explorer program [13]. ACE is less sensitive than PCF, but it detects well known amplification regions for chromosome 1, 8, and 17, in addition to an amplification increasing towards the telomere for chromosome 20, only detected in the Agilent platform. [file 1471-2164-9-379-S1.pdf]

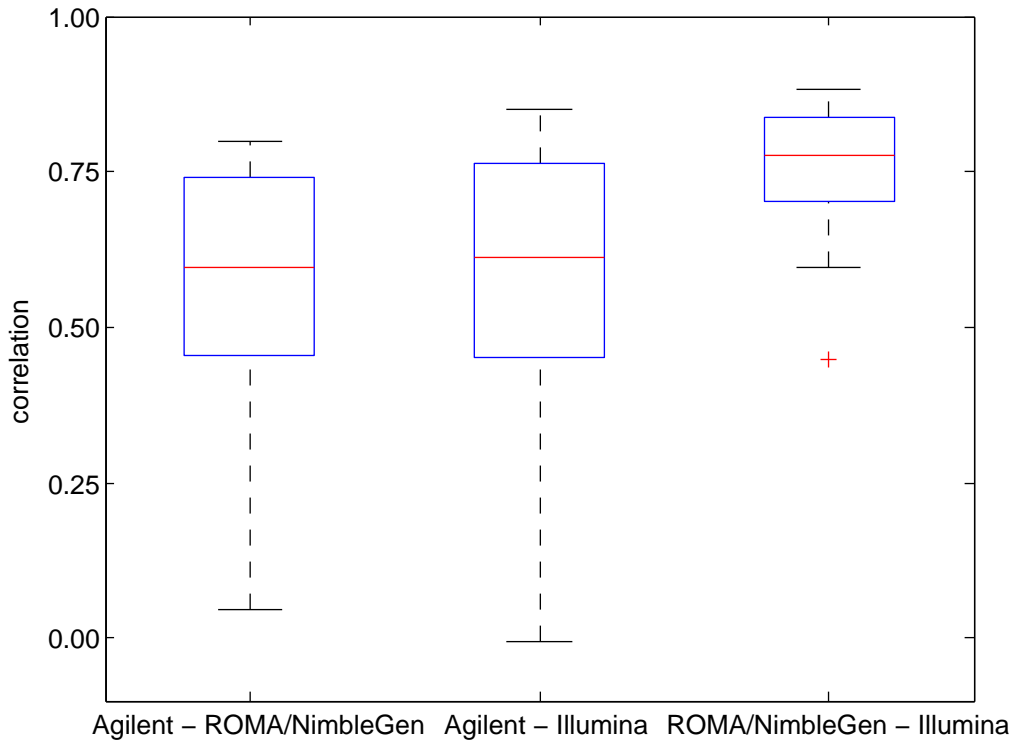

Supplement: Additional file 2 — Platform correlation. The three boxplots give the correlations between the platforms. Correlations are based on PCF values found for every array and for a regular genomic grid (see the Methods section on cross-platform copy number comparison), and the box plots shows the distribution of the 20 correlations found for every pair of platforms. [file 1471-2164-9-379-S2.pdf]

## Agilent 44k

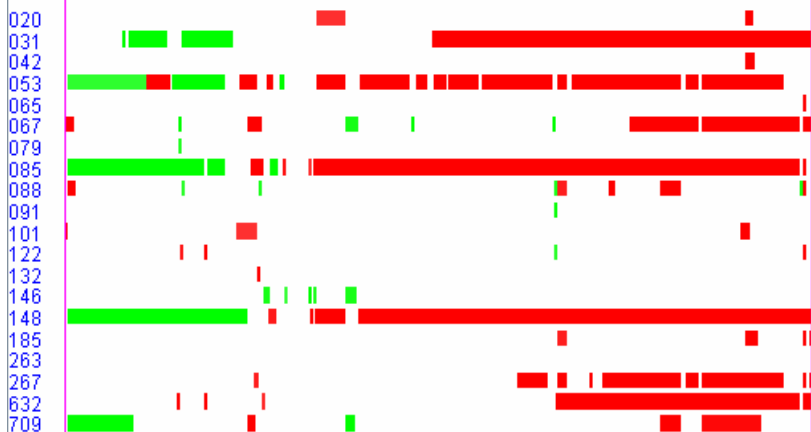

## ROMA/NimbleGen 82k

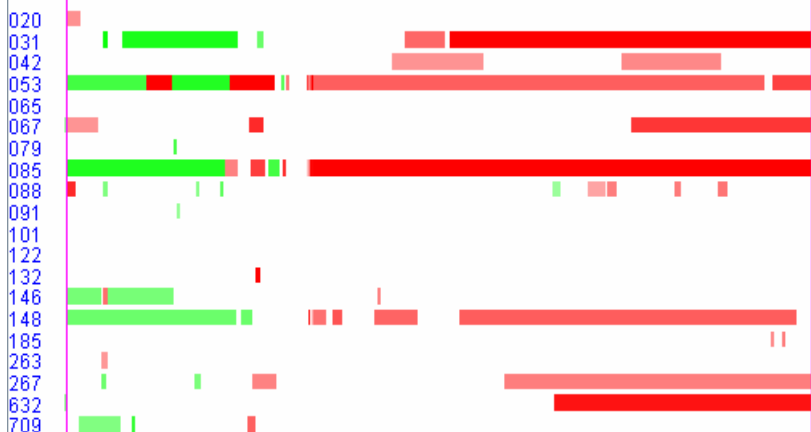

## Illumina 109k

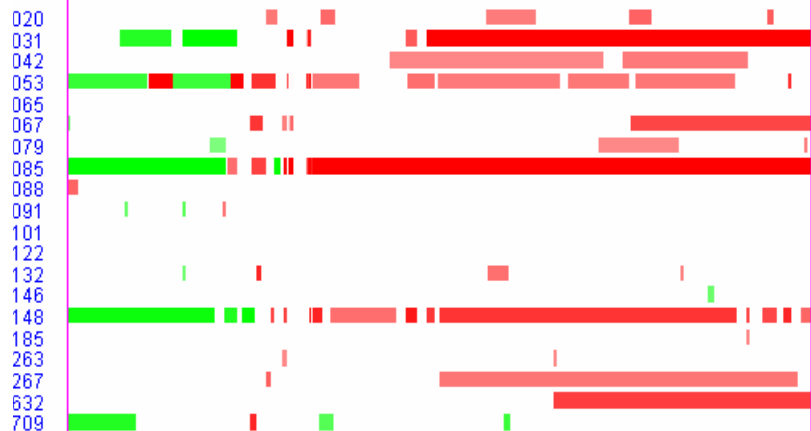

Supplement: Additional file 3 — Genomic aberration on chromosome 8 using PCF method. Single plot graphical views for each of the 20 tumor samples are shown using the Piecewise Constant Fit (PCF) method in the CGH-Explorer program for detection of copy number changes. Amplifications are highlighted in red and deletions are marked with green with color intensity coding from -0.5 to 0.5 and an overall high similarity are seen (see Methods). Despite wide similarities some differences are also detected. For example, sample 031 shows an interrupted deletion at the p arm for the Agilent and Illumina platforms missing in the ROMA/NimbleGen platform, further sample 042 shows a low copy number gain of several regions on 8q by the ROMA/NimbleGen and Illumina platforms, missing in the Agilent platform. [file 1471-2164-9-379-S3.pdf]

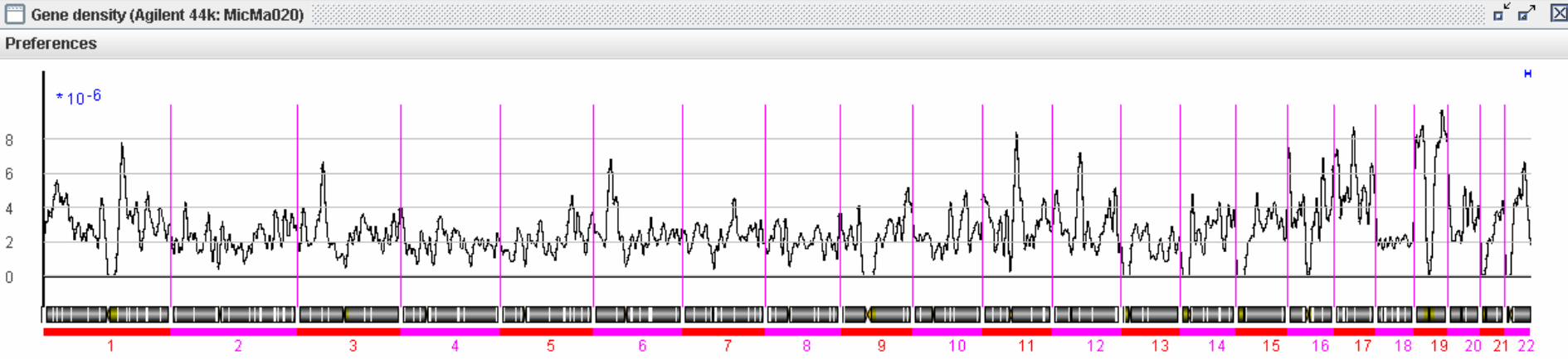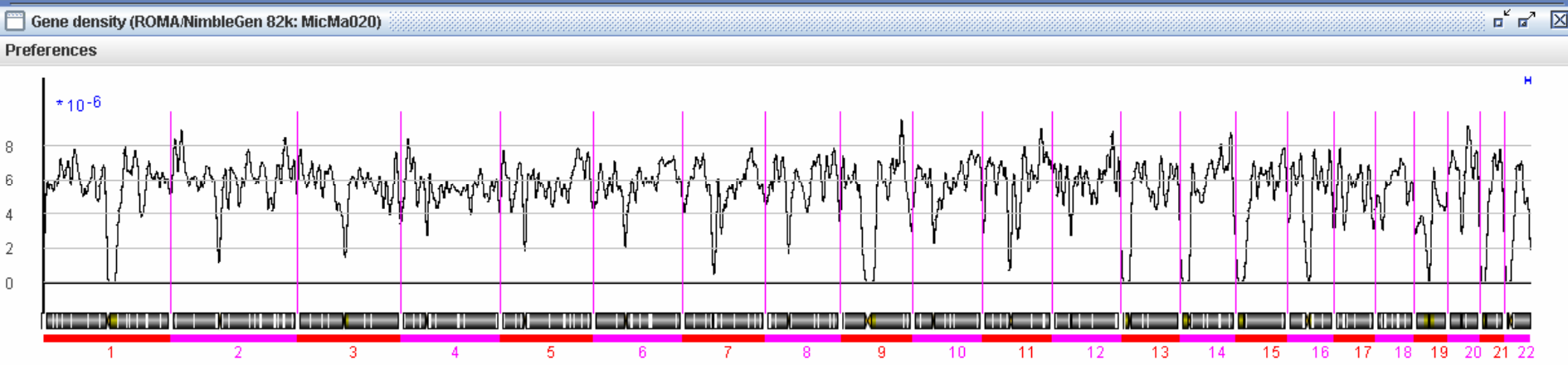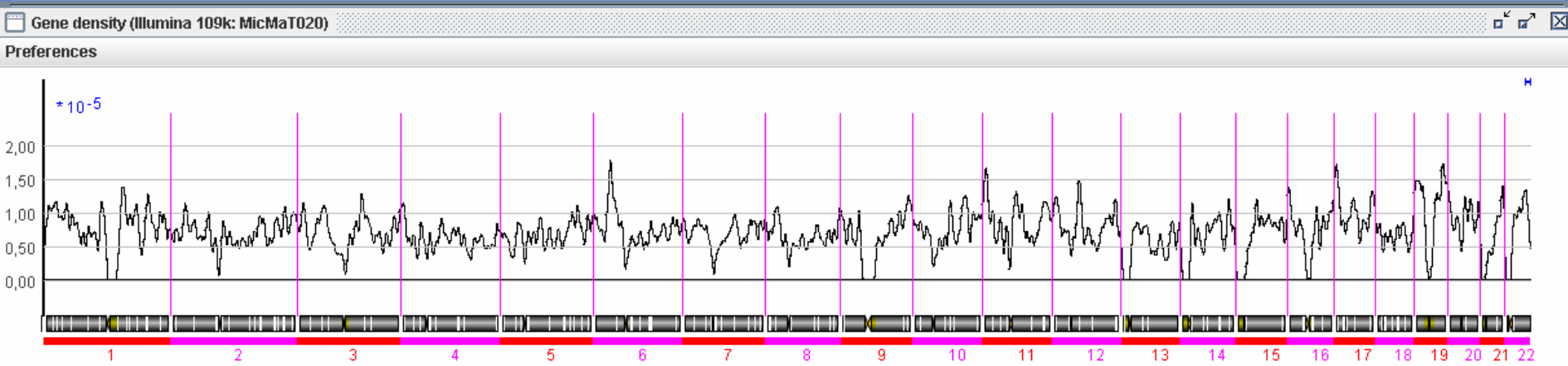

Supplement: Additional file 4 — Gene density plot for the whole genome. The distribution of probes along the complete genome is illustrated for the Agilent 44 k, ROMA/NimbleGen 82 k, and Illumina 109 k platforms with platform-dependent bandwidth selection (see Methods). Uneven distribution is seen for all platforms with areas of high density in certain chromosomes or chromosome arms. [file 1471-2164-9-379-S4.pdf]

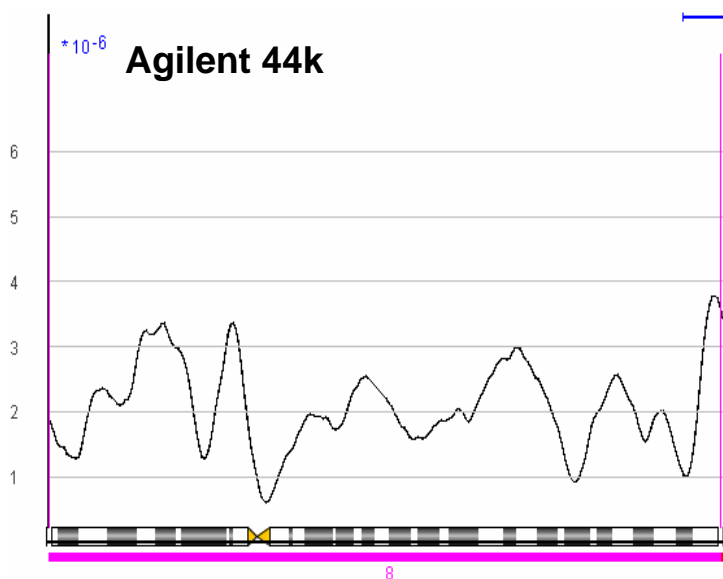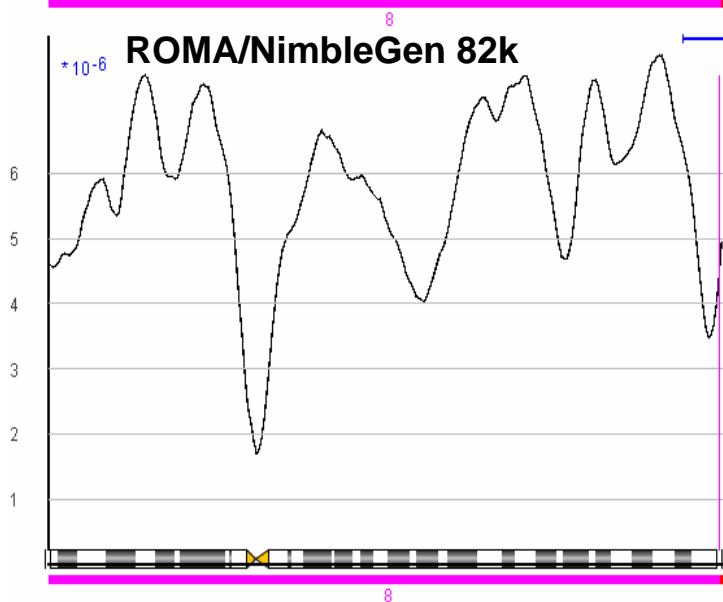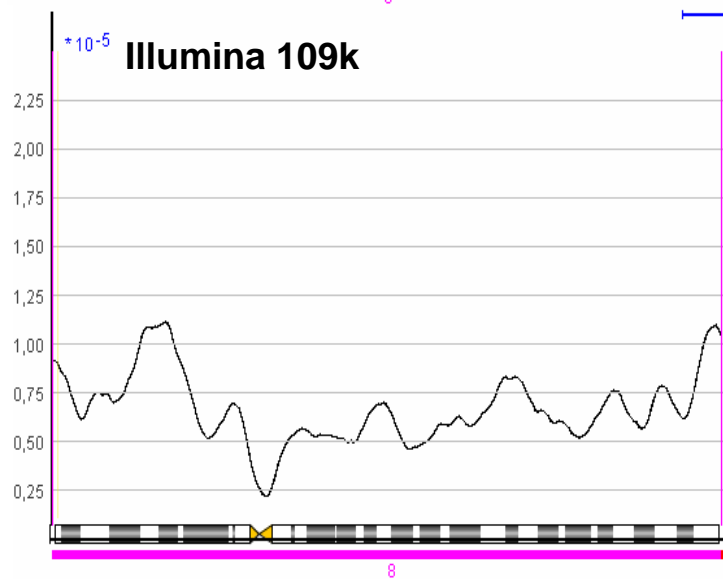

Supplement: Additional file 5 — Probe density plot for chromosome 8, The distribution of probes is illustrated for the complete genome for three different platforms with bandwidth size, Agilent = 10-6, ROMA/NimbleGen = 10-6, and Illumina = 10-5 for example chromosome 8 (gene density for the entire genome is presented in Additional file 6). [file 1471-2164-9-379-S5.pdf]
