# Supplementary material for: Comparison of the Agilent, ROMA/NimbleGen and Illumina platforms for classification of copy number alterations in human breast tumors
Source: BMC Genomics. 2008 Aug 8;9:379. doi: 10.1186/1471-2164-9-379 (PMC2547478; doi:10.1186/1471-2164-9-379)
Supplement: Additional file 13 — Details of standard protocols for the Agilent Human Genome CGH Microarray 44 k, ROMA/NimbleGen Representational Oligonucleotide Microarray 82 k, and Illumina SNP-CGH Human-1 109 k. [file 1471-2164-9-379-S13.doc]

**Additional file 13. Standard protocols for the Agilent Human Genome CGH Microarray 44k, ROMA/NimbleGen Representational Oligonucleotide Microarray 82k, and Illumina SNP-CGH Human-1 109k**

**Agilent standard protocol for Human Genome CGH Microarray 44k Microarray**

For each sample, 100 ng genomic DNA was amplified using phi29 DNA polymerase at 30 ºC, 16 hours over night. The following day the enzyme reaction was inactivated by incubation at 65 ºC for 10 minutes. The amplified DNA was digested using 50 units AluI and 50 units RsaI nucleases at a 2 hours incubation at 37 ºC. The amplified and digested DNA was purified using QIAprep Miniprep Kit (QIAGEN) according to manufacturer’s protocol, and quantified using a NanoDrop ND-1000 UV-Vis Spectrophotometer (NanoDrop Technologies). 10 μg amplified and digested DNA was labeled with Cy5-dUTP (PerkinElmer) using the BioPrime Labeling System (Invitrogen). The reference samples, female human genomic DNA (Promega), were labeled in parallel with Cy3-dUTP. Cy3 and Cy5 reactions were combined and purified using Microcon YM-30 Filters (Amicon), before loading onto the arrays and assembled in hybridization chambers according to manufacturer’s protocol. Arrays were incubated at 65 ºC for 40 hours on a Robbins Scientific oven with rotary motion (20 rpm). Hybridization chambers were taken out of the oven, arrays quickly disassembled in washing solution 1 (0.5xSSPE, 0.005% N-lauroylsarcosine) and placed in slide-rack submerged in fresh washing solution 1. Arrays were manually washed in batches of four; five minutes in washing solution 1, one minute in washing solution 2 (0.1xSSPE 0.005% N-lauroylsarcosine) at 37 ºC, one minute in 100% acetonitrile (Merck) and 30 seconds in stabilization & drying solution (Agilent Technologies). Arrays were protected from light until scanning. The arrays were scanned within 30 minutes after washing, using an Agilent Microarray Scanner. Male genomic DNA (Promega) was used as reference in all Agilent experiments.

**Standard protocol for the ROMA/NimbleGen 82k array**

For each sample, 1 μg tumor genomic DNA and 1 μg reference DNA (CHP-SKN-1 = 46, XY male) were separately digested using BglII (New England Biolabs) at 37 ºC overnight. EDTA was added to a final concentration of 15 mM and 2 μl yeast tRNA (10 mg/ml) was applied. The restriction fragments were purified using columns (Quiagen) and cutting was verified by agarose gel analysis. Adaptors (62 pM/μl 12-mer and 62 pM/μl 24-mer) were ligated with T4 Ligase (400 U/μl) and equal loading concentration was verified by agarose gel electrophoresis. DNA was amplified by adaptor mediated PCR using Taq polymerase (Perkin Elmer). The PCR reactions were purified using columns (Qiagen), followed by a final ethanol precipitation and then resupended in TE. PCR products were analyzed by 2% agarose gel electrophoresis to ensure adaptor removal and correct size representation of 200-1,200 bp. For each sample, 10 μg representation DNA was denatured by incubation at 100 ºC for 5 minutes followed by at least 5 min on ice. Samples were labeled with Cy5-dCTP or Cy3-dCTP using the Megaprime DNA labeling kit (Amersham Bioscience) and incubated for 2 hours at 37 ºC (each experiment was hybridized in duplicate for “dye swap” or “color reversal purposes). The labeled representations (Cy3 and Cy5) were combined, and Cot-1 DNA and yeast tRNA were added to the mixture to block unspecific hybridization. Low pH TE was added and the reaction was purified using Microcon YM-30 columns (Amicon). Hybridizations consisting of 25 μl hybridization solution (50% formamide, 5xSSC, 0.1% SDS) and 10 μl labeled DNA were denatured at 95 ºC for five minutes in a MJ Research Tetrad and pre-annealed at 37 ^o^C for 30 min. The solution was loaded on the arrays and hybridized under a coverslip at 42 ºC for 14 to16 hours in a 5xSSC and formamide moisturized hybridization oven. After hybridization, slides were quickly submerged in 0.2%SDS/0.2xSSC solution to remove cover slip, placed in a twirling slide rack and washed for one minute in fresh 0.2%SDS/0.2xSSC, one minute in 0.2xSSC and one minute in 0.05xSSC before they were dried by centrifugation at 600 rpm for 5 minutes.

**Illumina Human-1 Beadchip standard manufacturers protocol**

For each sample, 250 ng genomic DNA were denaturateded by incubation in 0.1N NaOH (Sigma-Aldrich) at room temperature for 10 minutes before adding the amplification master mix (WG#-AMM, Illumina). The amplification was performed in an Illumina Hyb Oven at 37 ºC for 20-24 hours. Amplified tumor DNA was fragmented by adding fragmentation mix (WG#-FRG, Illumina) and incubated at 37 ºC for 1 hour. Fragmented DNA was precipitated by adding precipitation solution (WG#-PA1, Illumina), 100% 2-propanol, incubated at 4 ºC for 30 minutes and further centrifuged at 4 ºC for 20 minutes. The supernatant was decanted by inverting the tubes, and DNA pellets were air dried at room temperature for one hour. DNA pellets were resuspended by adding resuspension solution (WG#-RA1, Illumina), incubated at 48 ºC for one hour and vortexed at 1,800 rpm for 1 minute. The BeadChips were washed and assembled in Tecan TeFlow chambers according to manufacturer’s protocol (Infinium I Assay). Resuspended DNA was denaturated at 95 ºC for 20 minutes prior to hybridization, and then dispensed onto BeadChips together with 100% molecular biology grade formamide (GF Fisher Scientific) and hybridization solution (WG#RA1, Illumina). Hybridization chambers were placed on rocker platform (5 rpm) inside the hybridization oven and incubated at 48 ºC for 16-24 hours. The TeFlow chambers (TFCs) were removed from the oven and placed into 44 ºC heated chamber racks. Wash solution WG#-RA1 (Illumina) was added to the TFC reservoirs and incubated for one minute, repeated four times. DNA targets were scored using an allele-specific primer extension reaction (ASPE) by incorporating multiple biotin labeled dNTP nucleotides into the appropriate allelic probe ^48^: XStain BeadChip solution 1 (WG#-XB1, Illumina) were added to each TFC reservoir and incubated for 10 minutes followed by a 5 minute incubation with XStain BeadChip solution 2 (WG#-XB2, Illumina). Further, extension master mix (WG#-EMM, Illumina) was added to the TFC reservoir and the BeadChips were incubated for 15 minutes, followed by two incubations with 95% formamide/1mM EDTA, one minute each. XStain BeadChip solution 3 (WG#-XB3, Illumina) was added to the reservoirs and incubated for one minute before staining. Labeling master mix (WG#-LMM, Illumina) was added to each TFC, incubated for 10 minutes before washing three times with XStain BeadChip solution 3, one minute each. Anti-stain master mix (WG#-ASM, Illumina) was added to each TFC and incubated for 10 minutes before washing three times with XStain BeadChip solution 3, one minute each. The whole labeling- and anti-stain procedure was repeated once followed by one last labeling- and washing step. BeadChips were disassembled from the TFC’s and immediately placed in wash rack submerged in WG#-PB1 solution. Slide racks were manually moved up and down ~20 times in the solution to wash BeadChips. The BeadChips were spun dry for one minute to avoid dried WG#-PB1 leaving any residue on the surface.
